# Perspective on direction of control: Cellular metabolism and macrophage polarization

**DOI:** 10.3389/fimmu.2022.918747

**Published:** 2022-09-08

**Authors:** Ronan Thibaut, Lucie Orliaguet, Tina Ejlalmanesh, Nicolas Venteclef, Fawaz Alzaid

**Affiliations:** ^1^ INSERM UMR-S1151, CNRS UMR-S8253, Université Paris Cité, Institut Necker Enfants Malades, Paris, France; ^2^ Dasman Diabetes Institute, Kuwait, Kuwait

**Keywords:** macrophage, metabolism, energetics, mitochondria, Inflammation

## Abstract

Macrophages are innate immune cells with high phenotypic plasticity. Depending on the microenvironmental cues they receive, they polarize on a spectrum with extremes being pro- or anti-inflammatory. As well as responses to microenvironmental cues, cellular metabolism is increasingly recognized as a key factor influencing macrophage function. While pro-inflammatory macrophages mostly use glycolysis to meet their energetic needs, anti-inflammatory macrophages heavily rely on mitochondrial respiration. The relationship between macrophage phenotype and macrophage metabolism is well established, however its precise directionality is still under question. Indeed, whether cellular metabolism *per se* influences macrophage phenotype or whether macrophage polarization dictates metabolic activity is an area of active research. In this short perspective article, we sought to shed light on this area. By modulating several metabolic pathways in bone marrow-derived macrophages, we show that disruption of cellular metabolism does *per se* influence cytokine secretion profile and expression of key inflammatory genes. Only some pathways seem to be involved in these processes, highlighting the need for specific metabolic functions in the regulation of macrophage phenotype. We thus demonstrate that the intact nature of cellular metabolism influences macrophage phenotype and function, addressing the directionality between these two aspects of macrophage biology.

## Introduction

Macrophages are innate immune cells that populate all tissues and have a number of homeostatic roles (e.g., removing dead cells and cellular debris, recycling iron). One of their main immune functions is to recognize and phagocytose pathogens (1). Following recognition, they also produce cytokines that can recruit and induce differentiation of monocytes and T cells. Additionally, they play an important role in tissue repair once the immune response has been terminated (1).

Macrophages can respond to a variety of molecular cues through activation of Toll-Like Receptors (TLRs), and will secrete cytokines accordingly. Following activation, they will transition from a quiescent state to an activated one. Depending on the cues they receive, they can adopt different activated phenotypes. Based on *in vitro* experiments, activated macrophages have been historically divided into M1 pro-inflammatory or M2 anti-inflammatory macrophages (2). Stimulating macrophages with lipopolysaccharide (LPS) and interferon-γ (IFN-γ) triggers their differentiation into M1 macrophages whereas interleukin 4 (IL4) and IL13 promote an M2 phenotype (3, 4).

**Figure 1:**
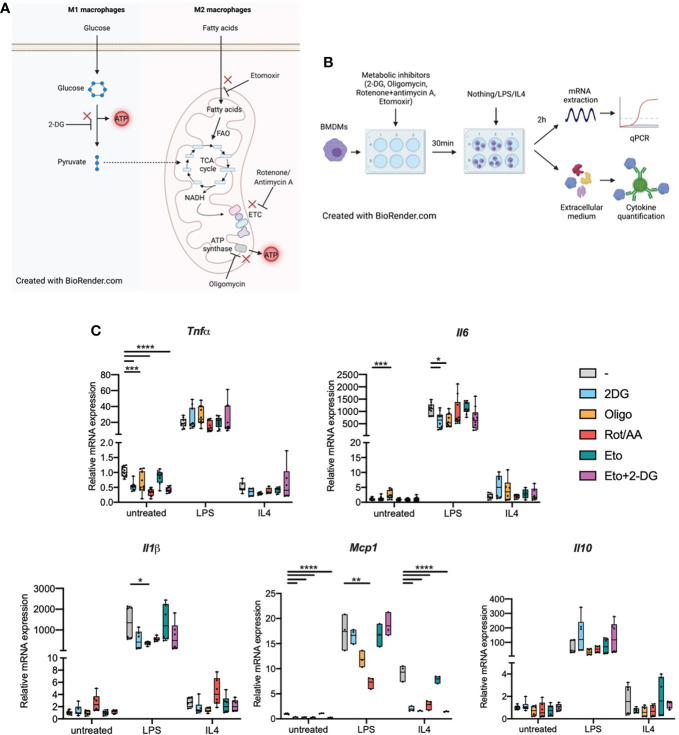
Metabolic inhibitors alter macrophage expression of inflammatory genes in response to LPS or IL4 **(A)**. M1 and M2 macrophage metabolism. FAO: fatty acid oxidation. ETC, Electron transport chain; TCA cycle, tricarboxylic acid cycle. **(B)** Experimental design: bone marrow-derived macrophages (BMDMs) were treated with metabolic inhibitors for 30 min. BMDMs were then either left untreated (M0 quiescent macrophages) or stimulated with LPS or IL4 to induce M1 and M2 polarizations respectively. Macrophage response was then assessed by quantifying cytokine concentration in the extracellular medium or through mRNA quantification by qPCR. **(C)** Relative mRNA expression for *Tnfa*, *Il6*, *Il1b*, *Mcp1* and *Il10* in quiescent BMDMs either left untreated or treated with 2-deoxyglucose (2-DG), oligomycin (Oligo), rotenone/antimycin A (Rot/AA), Etomoxir (Eto) or a combination of 2-DG and Etomoxir (Eto+2-DG). All points are shown in box plots with line at median. One-way ANOVA was used to determine statistical significance between groups within each stimulation condition (untreated, LPS, IL4) *p < 0.05, **p < 0.01, ***p < 0,001, ****p < 0,0001. Unless otherwise stated, differences between groups are not statistically significant. A and B Created with BioRender.com.

M1 and M2 macrophages display different metabolic properties. M1 macrophages have increased glucose uptake through the Hypoxia Inducible Factor 1α (HIF1α)-dependent upregulation of glucose transporters. This increased glucose uptake is essential to fuel glycolysis and thus produce ATP. M1 macrophages scarcely use mitochondrial respiration as they have targeted breaks in the tricarboxylic acid (TCA) cycle (**Figure 1A**) (5–7).

M2 macrophages, on the other hand, produce ATP mostly through mitochondrial respiration and have only limited glycolytic activity. Fueling the TCA cycle, M2 macrophages enhance their uptake of fatty acids. Fatty acid oxidation (FAO) provides Acetyl-CoA to a fully functional TCA cycle (8). TCA cycle-derived NADH is then used by the electron transport chain (ETC) to generate ATP through ATP synthase (**Figure 1A**) (7, 9).

*In vivo*, environmental factors that vary between different tissues or between homeostasis and disease are increasingly recognized as powerful dictators of macrophage metabolism. Macrophages in inflammatory conditions and inflammatory diseases are predominantly glycolytic. For example, macrophages in rheumatoid arthritis (RA) up-regulate the enolase enzyme, which stimulates production of pro-inflammatory cytokines, and show elevated succinate levels (10, 11). In diet-induced obesity, adipose tissue macrophages are hypermetabolic, up-regulating both glycolytic activity and mitochondrial respiration (12). Yet, these macrophages were long considered M1-like and are known to promote insulin resistance and metabolic syndrome (9). Such environmentally adapted profiles raise the question of precise directionality between macrophage metabolism and inflammatory profile.

Here, we use bone marrow-derived macrophages (BMDMs), with inhibitors of specific metabolic pathways to shed light on the direction of control between cellular metabolism and capacity to mount an inflammatory response. Using cytokine production as a functional read-out for macrophage phenotype, we show that impairing metabolic pathways drastically impacts macrophage phenotype. Some metabolic pathways have differential effects on cytokine secretion profile, suggesting that additional factors may directly influence macrophage phenotype in synergy with metabolic parameters.

## Results

### Biasing cellular metabolism alters inflammatory marker expression in quiescent macrophages

We first wondered whether biasing macrophage metabolism could per se influence macrophages in their quiescent state. Following treatment with inhibitors of glycolysis, mitochondrial respiration or both, we quantified transcription of inflammatory markers Tnfa, Il6, Il1b, Mcp1 and Il10 in M0 BMDMs or in BMDMs that were subsequently polarized towards a M1 or M2 phenotype (**Figure 1B**). When pre-treated with 2-deoxyglucose (2DG), a non-metabolizable glucose analog which inhibits glycolysis. Quiescent BMDMs showed decreased Tnfa and Mcp1 transcription compared to cells that were not pre-treated with 2DG, there was no change for Il6, Il1b or Il10 (**Figure 1C**). Inhibiting the mitochondrial ETC with Rotenone and antimycin A (Rot/AA) resulted in a similar decrease in Tnfa transcription. Oligomycin, an ATP synthase inhibitor, and etomoxir which inhibits carnitine palmitoyl transferase 1a (Cpt1a), FAO’s rate-limiting enzyme, did not show any significant effect on cytokine transcription (**Figure 1C**). The combination of etomoxir and 2DG (Eto+2DG) did not display any additive effect compared with the single inhibitor setting. These results suggest that cellular metabolism, in particular intact activity of glycolysis and mitochondrial ETC, is important to maintain macrophages in a quiescent state. However, the lack of impact of the different aforementioned metabolic inhibitors on Il6 and Il1b transcription suggests that metabolism only influences specific signals at quiescence. Conversely, some metabolic pathways resulted in increased transcription of inflammatory markers. For example, oligomycin results in increased Il6 expression in quiescent BMDMs (**Figure 1C**).

### Cellular metabolism influences macrophage transcriptional response to M1-like and M2-like polarizing stimuli

We next wondered about the impact of cellular metabolism on macrophage capacity to polarize or sensitivity to polarizing agents. BMDMs were pretreated with metabolic inhibitors then stimulated with bacterial LPS, the main TLR4 ligand known to induce M1-like polarization, or with IL4, a known stimulator of M2-like polarization (**Figure 1B**).

In cells pretreated with 2DG, LPS-induced expression of Il6 was attenuated. This effect was also observed in cells pretreated with oligomycin for both Il1b and Il6 (**Figure 1C**). Upon pretreatment with Rot/AA, LPS-induced expression of Mcp1 was decreased. Etomoxir pretreatment did not affect the response to LPS. These data suggest a limited contribution of FAO to the transcriptional response to LPS. (**Figure 1C**). These results indicate functional specificity in the requirement for glycolysis and ATP synthase activity in LPS-induced expression of Il6, Il1b and Mcp1 versus Tnfa and Il10 (i.e., Il6 expression, but not Tnfa, requires intact glycolytic flux and ATP synthase activity).

Additionally, we confirmed a relative state of quiescence in untreated BMDM through their increased expression of inflammatory markers in response to LPS and through surface expression of MHCII following long-term treatment with LPS (**Figure S1**).

Following IL4 stimulation, few cytokines were affected at the transcriptional level. Only Mcp1 expression was affected (**Figure 1C**). Inhibiting any metabolic pathway other than FAO alone strongly decreased Mcp1 transcription in response to IL4. This indicates that FAO does not significantly contribute to the Mcp1 transcriptional response and while glycolysis did not seem to play any role in Mcp1 up-regulation following LPS stimulation, it was pivotal in the response to IL4.

### Cellular metabolism influences macrophage cytokine secretion in response to an inflammatory challenge

**Figure 2:**
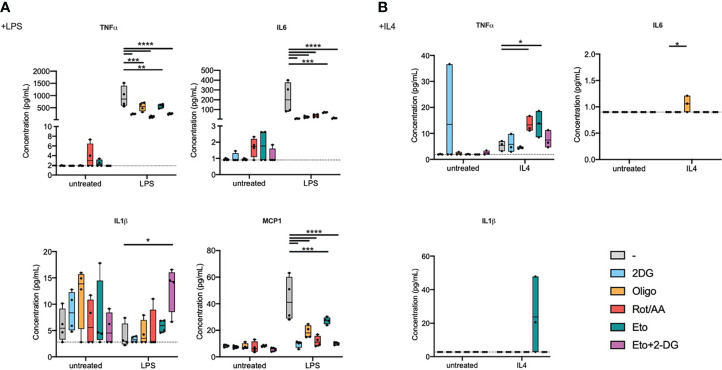
Metabolic inhibitors alter macrophage secretory profile in response to LPS or IL4. Bone marrow-derived macrophages (BMDMs) were pretreated with metabolic inhibitors for 30 min before being stimulated with M1 (LPS) or M2 (IL4) stimulus or left untreated for 2 h. Macrophage response was then assessed by quantifying cytokine concentration in the extracellular medium. **(A)** Concentration of TNFα, IL6, IL1β and MCP1 in cell culture media of BMDMs pretreated with 2-deoxyglucose (2-DG), oligomycin (Oligo), rotenone/antimycin A (Rot/AA), Etomoxir (Eto) or a combination of 2-DG and Etomoxir (Eto+2-DG) and then stimulated with LPS for 2h. **(B)** Concentration of TNFα and MCP1 in cell culture media of BMDMs pretreated with 2DG, Oligo, Rot/AA, Eto or Eto+2-DG and then stimulated with IL4 for 2h. All points are shown in box plots with line at median. The dashed line indicates detection limit of the assay. One-way ANOVA was used to determine statistical significance between groups within each stimulation condition (untreated, LPS, IL4) *p < 0.05, **p < 0.01, ***p < 0,001, ****p < 0,0001. Unless otherwise stated, differences between groups are not statistically significant.

As a functional readout, focusing on proinflammatory signalling, we measured cytokine secretion into the extracellular medium (**Figure 2A**). Inhibiting any of the aforementioned metabolic pathways resulted in decreased IL6, TNFα, and MCP1 secretion in response to LPS. Metabolic inhibition, without supplementary stimulation, did not induce significant changes in IL1β secretion. We did however observe a significant increase in IL1β secretion during LPS stimulation in cells that were pre-treated with Eto+2-DG, compared to other pretreatments (**Figure 2A**). While LPS stimulation alone is not expected to induce significant inflammasome activation, it is possible that the Eto+2DG combination, with LPS, induces cellular stresses that contribute to IL1β secretion.

In response to LPS, inhibiting glycolysis had the most dramatic and consistent effect across measured cytokines. Inhibiting the mitochondrial ETC at multiple levels, NADH:ubiquinone oxidoreductase, coenzyme Q-cytochrome c reductase or ATP synthase (Complexes I, III or V), comparably attenuated cytokine secretion. Inhibiting FAO had the least pronounced effect on cytokine secretion, even if it still decreased IL6 and MCP1 secretion (**Figure 2A**).

In response to IL4 stimulation, most inflammatory cytokines were secreted at low concentrations that were close to, or below, the detection limit for most samples (**Figure 2B**). TNFα was the exception that increased with IL4 treatment and its expression was potentiated upon pretreatment with etomoxir or with Rot/AA (**Figure 2B**). MCP1 expression showed a tendency to a decrease by oligomycin, Rot/AA and Eto+2-DG that did not however reach statistical significance (**Figure S2**).

These results suggest that targeting metabolic pathways alters macrophage secretory capacity in response to LPS or to IL4. Glycolysis and the mitochondrial ETC are instrumental in cytokine secretion in response to TLR4 ligation, while FAO contributes to a more limited extent. Elements of FAO and OXPHOS do however contribute to regulating TNFα expression in response to IL4. Overall, these findings show that metabolism is important for macrophage secretory profile. However, only specific metabolic pathways are capable of influencing transcriptional profile. Indeed, macrophage cellular metabolism greatly impacts macrophage capacity to polarize and specific metabolic pathways have differential roles in supporting an M1 or M2 phenotype (**Table 1**).

**Table 1:** Summary of effects of metabolic inhibitor pretreatments on gene expression and cytokine concentration in media from macrophages.

	M0 macrophages (unstimulated)					M1 macrophages (LPS)					M2 macrophages (IL4)				
	2-DG	Oligo	Rot/AA	Eto	Eto+2-DG	2-DG	Oligo	Rot/AA	Eto	Eto+2-DG	2-DG	Oligo	Rot/AA	Eto	Eto+2-DG
*Tnfa*	down	down	down	=	down	=	=	=	=	=	=	=	=	=	=
*Il6*	=	up	=	=	=	down	down	=	=	=	=	=	=	=	=
*Il1b*	=	down	=	=	=	=	down	=	=	=	=	=	=	=	=
*Mcp1*	down	down	down	=	down	=	=	down	=	=	down	down	down	=	down
*Il10*	=	=	=	=	=	=	=	=	=	=	=	=	=	=	=
TNFα	=	=	=	=	=	down	down	down	=	down	=	=	=	=	=
IL6	=	=	=	=	=	down	down	down	down	down	=	=	=	=	=
IL1β	=	=	=	=	=	=	=	=	=	up	=	=	=	=	=
MCP1	=	=	=	=	=	down	down	down	down	down	=	=	=	=	=

Bone marrow-derived macrophages were pretreated with metabolic inhibitors for 30 min. Macrophages were either left untreated (M0 quiescent macrophages) or stimulated with LPS or IL4 to induce M1 and M2 polarizations respectively. Pretreatments were 2-deoxyglucose (2-DG), oligomycin (Oligo), rotenone/antimycin A (Rot/AA), Etomoxir (Eto) or a combination of 2-DG and Etomoxir (Eto+2-DG). Direction of change indicated is relative to the non-pretreated control in each polarization state. Gene expression results are indicated in lower case italics and proteins secreted are in upper case.

## Discussion

Metabolism is tightly linked to macrophage polarization as M1 macrophages are predominantly glycolytic while M2 macrophages rely heavily on mitochondria. Here, we studied the impact of macrophage cellular metabolism on macrophage phenotype. We show that macrophage polarization depends on cellular metabolic activities and more particularly on some pivotal metabolic pathways like glycolysis. Interestingly, this dependency is functionally specific with respect to the cytokines affected and alters secretory capacity to a greater extent than transcription.

Additionally, we show that transcription of some cytokines was unexpectedly boosted by metabolic inhibition. It is possible that, following inhibition of a given pathway, metabolites used by this pathway are redirected towards other pathways in which activity is increased. This could be a mechanism of adaptation to the metabolically challenging environment. Such compensatory mechanisms have already been described in monocytes, in which IL6 production is quickly restored after depriving monocytes of ATP and glucose (13).

The impact of cellular metabolism on quiescent macrophage polarization differs between M1 and M2 macrophages. The impact of metabolic inhibition on M2 differentiation seems to be more limited than on M1 differentiation. This is of particular interest in some contexts like metabolic syndrome. In this disease, macrophages face changes in metabolic substrate availability, such as lipid overload. Moreover, in treated individuals, macrophages are also exposed to metabolically active therapeutic agents. Macrophages can accumulate in metabolic organs, adipose tissue and the liver, and exhibit an M1-like phenotype which is central to metabolic decline and the development of insulin resistance (9, 14, 15). Thus, our data suggest that changes in the metabolic environment of the cells could lead to changes in macrophage metabolic status and subsequently in macrophage phenotype. Alternatively, in therapeutic perspectives, altering availability of substrates or functioning of metabolic pathways can profoundly impact macrophage secretory profile. The question remains in defining at-risk groups and controlling cell-specific delivery of metabolically acting drugs. Future preclinical work will shed light on the feasibility of such approaches.

The M1/M2 classification has been challenged since it was first proposed. It poorly recapitulates the diversity of macrophage subpopulations found *in vivo*. Macrophages display great phenotypic and functional diversity depending on the tissues they populate, their ontogeny, or their states in health and disease (16). Such specificity could also arise in macrophage reliance on metabolic pathways or on adaptability of cellular metabolism. Additionally, recent studies have uncovered diversity in macrophage populations of a given tissue. For instance, 17 showed the existence of two subpopulations of macrophages that are conserved across several tissues and are localized in distinct tissular niches (17). Whether such subpopulations share common metabolic features or whether their different localizations also translate into different metabolic activities remains largely unknown. Differences in metabolic status between tissue-resident and monocyte-derived macrophages are still under investigation.

Future work could decipher the metabolic diversity of macrophages *in vivo*, adding mechanistic insight and rationale for redeployment of metabolically active therapeutics. Until recently, such investigations were considered technically challenging. However, techniques allowing single-cell resolution in analysis, e.g., SCENITH (18), are gaining accessibility and bring us closer to overcoming the technical challenges encountered when studying cellular heterogeneity across tissues, both in health and disease.

Macrophage polarization is known to play an active role in a number of diseases such as metabolic syndrome, as mentioned above, auto-immune diseases or cancer. While M1-like macrophages are important drivers of the progression of metabolic diseases, a phenotypic shift from M1-like to M2-like macrophages is known to favor cancer progression and eventually metastasis by dampening the anti-tumor immune response (19). Macrophage metabolism could therefore become an interesting therapeutic target in such diseases. Indeed, targeting relevant metabolic pathways could allow reprogramming of macrophage polarization and thus improve the disease. In addition, macrophages themselves are more and more considered as potential therapeutic agents, for example as chimeric antigen receptor (CAR)-macrophages targeting and phagocytosing tumor cells (20, 21). Combinations of such therapeutic macrophages with metabolic drugs aimed at modulating their phenotype could be powerful tools in a wide range of diseases.

## Methods

### BMDMs production and stimulation

C57BL/6J mice were bred and housed at the “Centre d’Explorations Fonctionnelles” bdof Sorbonne University (UMS-28) on a 12h day/night cycle. All experiments were approved by the French ethical board (Paris-Sorbonne University, Charles Darwin N°5, 01026.02) and conducted in agreement with all French regulations and guidelines. Tibias and femurs from C57BL/6J mice were collected. Bone marrow cells were recovered by flushing the bones with PBS. After red blood cell lysis, cells were plated in culture-treated 12- or 24-well plates at a concentration of 1x106/mL in BMDM medium (DMEM with GlutaMAX supplemented with 10% FCS, 30% L929-conditioned medium and 100U/mL Penicilin, 100µg/mL streptomycin). L929 cell line produces high amounts of macrophage-colony stimulating factor and other proteins stimulating macrophage differentiation (22). L929-conditioned medium thus allows differentiation of hematopoietic stem cells into BMDMs. Culture medium was renewed every two days.

After 6 to 7 days of differentiation, BMDMs were pre-treated for 30 min with either 10mM 2-DG (D8375), 10µM rotenone (R8875), 5µM antimycin A (A8674), 10µM oligomycin (O4876), 10µM etomoxir (E1905) or a combination of several of these drugs for 30min. The cells were then stimulated with LPS (10ng/ml) (L2630) or IL4 (10ng/mL) (130-094-061, Miltenyi Biotech) for 2h in continued presence of metabolic inhibitors. Unless otherwise stated, all compounds were purchased from Sigma Aldrich.

### Quantification of cytokine transcription

mRNAs were purified using either Qiagen RNeasy or Macherey-Nagel Nucleospin RNA kits, according to manufacturer’s instruction.

mRNAs were retrotranscribed into cDNAs using M-MLV Reverse Transcriptase kit (Promega). Quantitative RT-PCRs were performed with MESA green mastermix (Eurogentec) and target-specific primers using QuantStudio 3 Real-Time PCR Systems (ThermoFisher Scientific). 18S RNA was used for normalization of mRNA levels. The DNA sequences of primers used for qPCR are listed in **Table 2**.

**Table 2:** Sequences of DNA primers used in RT-qPCR reactions.

	Forward	Reverse
*18S*	GGGAGCCTGAGAAACGGC	GGGTCGGGAGTGGGTAATTT
*Il6*	TACCACTTCACAAGTCGGAGGC	CTGCAAGTGCATCATCGTTGTT
*Tnfa*	CCACCACGCTCTTCTGTCTA	CACTTGGTGGTTTGCTACGA
*Mcp1*	GGGCCTGCTGTTCACAGTT	CCAGCCTACTCATTGGGAT
*Il10*	GCTGGACAACATACTGCTAACC	ATTTCCGATAAGGCTTGGCAA
*Il1b*	GCAACTGTTCCTGAACTCAACT	ATCTTTTGGGGTCCGTCAACT

### Quantification of cytokine secretion

Culture medium from stimulated BMDMs was recovered and cytokine quantification was performed using BioLegend LegendPlex kit according to manufacturer’s instructions. Data were analyzed using Qognit software. Cytokines secreted by LPS-stimulated cells and cytokines secreted by IL4-stimulated cells were quantified using two lots of the LegendPlex kit.

### Statistical analysis

Statistical analysis was performed with GraphPad Prism (La Jolla, CA, USA). Data are presented as mean ± SEM. Comparison between groups was performed with either one-way ANOVA followed by Tukey’s test or two-way ANOVA followed by Dunnett’s multiple comparison test.

## Data availability statement

Source data will be made available by the authors upon reasonable request.

## Author contributions

RT, LO and FA designed the study. RT, LO and TE produced and analyzed experimental data. RT and FA wrote the manuscript. LO, TE and NV contributed in manuscript writing. All authors contributed to the article and approved the submitted version.

## Funding

This work was supported by the French National Research Agency (Agence Nationale de la Recherche; ANR) ANR-JCJC grant for the MitoFLAME Project ANR-19-CE14-0005 to FA. RT was supported by a grant from the European Foundation for the Study of Diabetes (EFSD). LO was supported by Fondation de la Recherche Médicale (FDT202106013230).

## Conflict of interest

The authors declare that the research was conducted in the absence of any commercial or financial relationships that could be construed as a potential conflict of interest.

## Publisher’s note

All claims expressed in this article are solely those of the authors and do not necessarily represent those of their affiliated organizations, or those of the publisher, the editors and the reviewers. Any product that may be evaluated in this article, or claim that may be made by its manufacturer, is not guaranteed or endorsed by the publisher.

## Supplementary material

Supplementary Figure 1 Macrophage expression profile at quiescence in response to metabolic inhibitors. Bone marrow-derived macrophages (BMDMs) were pretreated with metabolic inhibitors for 30 min and left untreated in clean cell culture media for 2 h after. Surface expression levels of MHCII by flow cytometry in BMDM that are unstimulated or stimulated with LPS for 2 h or 24 h. All points are shown in box plots with line at median. ns not significant, * p<0.05, *** p<0,001, **** p<0,0001. Unless otherwise stated, differences between groups are not statistically significant.

Supplementary Figure 2 MCP1 secretion by IL-4-stimulated BMDMs. Concentration of MCP1 in cell culture media of BMDMs pretreated with 2DG, Oligo, Rot/AA, Eto or Eto+2-DG and then stimulated with IL4 for 2h. All points are shown in box plots with line at median. One-way ANOVA was used to determine statistical significance between groups within each stimulation condition (untreated, LPS, IL4) * p<0.05, *** p<0,001, **** p<0,0001. Unless otherwise stated, differences between groups are not statistically significant.
