# Supplementary figures and images for: Perspective on direction of control: Cellular metabolism and macrophage polarization
Source: Front Immunol. 2022 Sep 8;13:918747. doi: 10.3389/fimmu.2022.918747 (PMC9493491; doi:10.3389/fimmu.2022.918747)

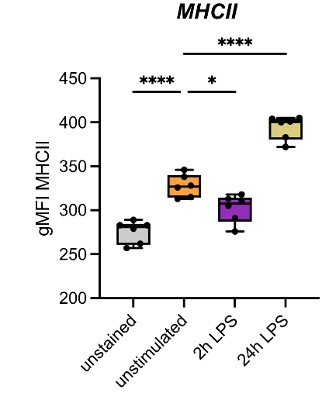

Supplement: Supplementary Figure 1 — Macrophage expression profile at quiescence in response to metabolic inhibitors. Bone marrow-derived macrophages (BMDMs) were pretreated with metabolic inhibitors for 30 min and left untreated in clean cell culture media for 2 h after. Surface expression levels of MHCII by flow cytometry in BMDM that are unstimulated or stimulated with LPS for 2 h or 24 h. All points are shown in box plots with line at median. ns not significant, * p<0.05, *** p<0,001, **** p<0,0001. Unless otherwise stated, differences between groups are not statistically significant. [file Image_1.jpeg]

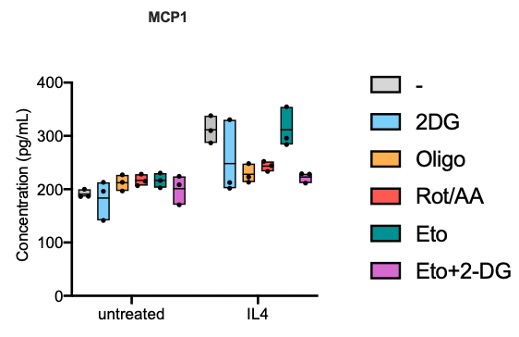

Supplement: Supplementary Figure 2 — MCP1 secretion by IL-4-stimulated BMDMs. Concentration of MCP1 in cell culture media of BMDMs pretreated with 2DG, Oligo, Rot/AA, Eto or Eto+2-DG and then stimulated with IL4 for 2h. All points are shown in box plots with line at median. One-way ANOVA was used to determine statistical significance between groups within each stimulation condition (untreated, LPS, IL4) * p<0.05, *** p<0,001, **** p<0,0001. Unless otherwise stated, differences between groups are not statistically significant. [file Image_2.jpeg]
